# Endoscopic Optical Coherence Tomography for Assessing Inhalation Airway Injury: A Technical Review

**DOI:** 10.4172/2161-119X.1000366

**Published:** 2019-04-04

**Authors:** Yusi Miao, Matthew Brenner, Zhongping Chen

**Affiliations:** Beckman Laser Institute, University of California, Irvine, CA, USA

**Keywords:** Inhalation injury, Endoscopic optical coherence tomography, Airway monitoring, Airway obstruction

## Abstract

Diagnosis of inhalation injury has been clinically challenging. Currently, assessment of inhalation injury relies on subjective clinical exams and bronchoscopy, which provides little understanding of tissue conditions and results in limited prognostics. Endoscopic Optical coherence tomography (OCT) technology has been recently utilized in the airway for direct assessment of respiratory tract disorders and injuries. Endoscopic OCT is capable of capturing high-resolution images of tissue morphology 1-3 mm beneath the surface as well as the complex 3D anatomical shape. Previous studies indicate that changes in airway histopathology can be found in the OCT image almost immediately after inhalation of smoke and other toxic chemicals, which correlates well with histology and pulmonary function tests. This review summarizes the recent development of endoscopic OCT technology for airway imaging, current uses of OCT for inhalation injury, and possible future directions.

## Introduction

Despite the recent advancements in therapeutics and counteragents, mortality and morbidity from inhalation injury remain high [1,2]. Inhalation of toxins often affects multiple airway sites at different times, making diagnosis and prediction of injuries challenging: it often results in a combination of 1) thermal damage in the upper airway, 2) chemical injury that affects both the upper and lower respiratory tract, and 3) systematic effects from toxins, such as CO, CN, and cyanide [3]. Heated air from fires can cause thermal injury to the upper airway, which leads to swelling and edema of the tongue, epiglottis, and aryepiglottic folds [4]. If significant thermal damage is suspected, continuous monitoring is required to assess the need for ventilation. Chemical irritants can affect airway at different levels depending on their chemical characteristics and water solubility, with smaller particles and less water-soluble gases generally affect the lower respiratory tract. Inhalation of chemical irritants often results in mucosa damage, leading to ciliastasis, epithelium denudation, sloughing, airway obstruction, and pulmonary edema [5,6]. The number of substances that can cause severe lung damage continues to expand in both occupational and domestic settings. Besides direct and immediate effects, in many cases, inhalation injury is accompanied by inflammatory responses that can prolong the ventilation period and increase the risk of acute respiratory distress syndrome. Therefore, accurate diagnosis and continuous monitoring of affected airway tissue are crucial. Monitoring of airway injuries has been challenging due to its anatomical location. Currently, the clinical diagnosis and monitoring of airway inhalation injury rely on subjective clinical exams and bronchoscopic findings. However, only the superficial tissue can be seen using a bronchoscope, and the measurements from clinical exams are subjective. Computerized tomographic (CT) scanning has recently been proposed for quantitative airway injury assessment [7]. A three-dimensional reconstruction of the airway from a chest CT can provide information of airway narrowing [8]. However, the CT scan does not provide enough spatial resolution to identify early tissue damage, such as hyperemia, sloughing, necrosis, and inflammation, and thus, CT cannot provide an accurate assessment of inhalation injury by itself [9]. In addition, repeated CT measurement expose the patient to the risks of ionizing radiation. Ultrasound has been proposed as a non-invasive point-of-care imaging tool for assessment of upper airway health [10].Unlike CT and Magnetic resonance imaging (MRI), ultrasound imaging requires minimal training and is a rapid diagnostic tool that does not require sedation. It has been used to detect tracheal wall thickness of a patient with smoke inhalation injury, with accuracy comparable to those of CT [11,12]. However, ultrasound alone cannot provide an accurate diagnosis of inhalation injury due to its limited imaging area and relatively low spatial resolution. Most importantly, ultrasound must be performed in the contact mode in order to visualize structures below the airway surface, which is not practical in the larger airways. Currently, neither CT nor ultrasound imaging has the capability to resolve early changes in the mucosa layer due to chemical irritants and edema. Therefore, a medical imaging tool that can detect early signs of airway injuries as well as monitor the recovery process is required to improve patient outcome.

Optical coherence tomography (OCT) provides non-invasive and real-time visualization of biological tissue 1-3 mm beneath the surface with virtually histologic-level resolution. OCT has previously been used in ophthalmology, cardiology, and dermatology to improve patient outcome. Recent advancements in a high-speed swept-source laser allows 3D volumetric scanning of tissue in real-time during procedural settings [13]. Since the imaging part of OCT can be made with fiber optics, OCT can be made into miniature rigid or flexible endoscopic probes to visualize internal organs that were previously hard to reach, similar to fiber optic bronchoscopy. Endoscopic OCT has started to be utilized in humans in clinical settings to visualize the respiratory tract [14-16] and gastrointestinal tract [17,18].

While some previous studies discussed the development of endoscopic OCT techniques for airway and lung imaging, few studies have focused on the assessment and monitoring of inhalation injury [19,20]. Therefore, this review focuses on the recent development and applications of endoscopic airway OCT for assessing inhalation injuries and discuss the potential use of OCT in diagnosis-specific assessment and treatment.

## Development of Endoscopic OCT for airway imaging

Several studies have used endoscopic OCT to study airway diseases and disorders [14-16,21,22]. Endoscopic OCT commonly employs fiber optic-based imaging techniques that can be combined with other imaging modalities, such as bronchoscopy, to examine internal organs. Tearny et al., reported the first application of an endoscopic OCT study in an in vivo rabbit trachea using a Time-domain (TD) OCT system [23]. With the advancement of the high-speed sweeping laser, the imaging speed and sensitivity of endoscopic OCT has improved in recent years [14,16,24,25].

### Early airway studies using TD OCT

In early studies, TD-OCT has been used to image the airway [23,26]. In TD-OCT, the optical path length of the reference arm in an interferometer is mechanically varied to get signals at different depths of the sample. Therefore, the imaging range of TD-OCT is determined by the moving range of the reference arm. The main limitation of TD-OCT is the scanning speed. In the case of endoscopic imaging, slower scanning speed can result in motion artifact and lengthen diagnostic procedure time. To achieve real-time imaging with TD-OCT, several groups adopted a rapid scanning optical delay line in the reference arm to scan across the sample. In this setup, the group delay was changed rapidly by moving a mirror mounted on a galvanometer, allowing 500 A-line scans per second with a 36 mm scanning distance [27]. However, there is a trade-off between the scanning speed and sensitivity in TD-OCT so the fast scanning will result in lower signal-to-noise ratio.

### Development of Fourier domain OCT for airway imaging

With the development of Fourier domain OCT (FD-OCT), the speed and sensitivity of endoscopic imaging have significantly improved. Since FD-OCT obtains depth information of the sample simultaneously through a wavelength-sweeping laser or a spectrometer and a broadband laser rather than mechanically scanning the reference arm, the imaging speed can be much faster than TD-OCT. However, the main drawback of FD-OCT is the finite imaging range. Typically imaging the entire airway lumen requires a 25 mm imaging range. Jun et al., overcame the short imaging range by implementing a phase modulator and effectively doubled the imaging range [28]. More recently, Jing et al. applied a vertical-cavity surface-emitting laser (VCSEL) based swept source laser and achieved long-range OCT imaging with much higher sensitivity by taking advantage of its narrow instantaneous pulse width of light source [14]. Super-high-speed endoscopic imaging using a Fourier domain mode lock laser has been demonstrated with an MHz scanning rate [24]. In addition to the speed, the axial resolution has been improved up to 1 um by using a broad bandwidth light source such as a supercontinuum [29,30].

### Different types of endoscopic imaging probes for airway application

**Figure 1:**
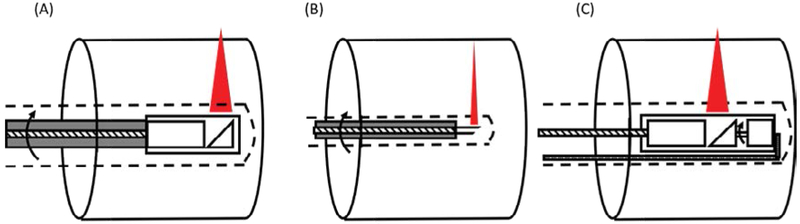
Three types of endoscopic probes commonly used in airway imaging: (A) proximal rotational probe based on a GRIN lens, (B) proximal rotational probe based on an all-fiber optic design, and (C) distal rotational probe based on micro-motor. All endoscopic probes are protected with a disposable plastic catheter (dotted line) to prevent direct contact with tissue.

Most commonly used scanning schemes for endoscopic OCT imaging utilize proximal rotation or distal rotation driving mechanisms. The proximal rotational endoscopic probe utilizes a fiber optic rotary junction to drive the imaging probe externally. In addition to the flexible sheath that prevents contact of the probe with tissue, the entire probe is protected with a metal housing or a torque coil to reduce the friction during the high-speed rotation. For scanning large animals and the human airway, a GRIN lens-based flexible endoscopic probe is commonly used (Figure 1A). The distal end of the probe consists of a mirror, GRIN lens, and a spacer. The focusing distance of the probe can be adjusted based on the scanning site and the diameter of the airway. The probe diameter ranges from 2 mm to 0.9 mm. In some applications, even a smaller endoscopic probe is required, such as for imaging the respiratory tract of a small animal or terminal bronchus of a larger animal. In such cases, a Graded index (GRIN) fibers [31], a ball lens [32], or large core fibers [33,34] can be used instead of a lens to fabricate an endoscopic probe as small as an optical fiber, typically less than 250 um (Figure 1B). The fiber end is polished at a critical angle in order to reflect OCT light without a mirror. The advantages of the proximal rotational scheme are the simplicity of fabrication and the ability to be able to miniaturize the probe. However, the probe rotational speed is limited since the entire probe rotates, increasing the time to acquire the full 3D scanning image. In addition, bending the probe can cause Non-uniform rotational distortion (NURD). The distal rotational probe typically utilizes MEMS scanning mechanics (Figure 1C) [14,35]. A miniature motor is placed at the distal portion of the probe next to the focusing lens. Since the moving unit is limited to the probe tip, the probe bending does not cause NURD and the rotation speed can be higher, making this method more suitable for high-speed scanning. However, the micromotor makes the probe size slightly larger than the proximal scanning probe. In addition, the electronic wire for controlling the motor will partially block the OCT light and cast a shadow to the OCT image.

## OCT for Assessing Inhalation Airway Injury

OCT has been used in numerous inhalation injury studies to assess tissue damage and predict outcome from toxic gas exposure [36-39]. Epithelium and mucosa layer thickness are the main parameters that have been used to assess early tissue response to the inhaled substance. It was demonstrated that the mucosa thickness reflects the early changes in the tissue and OCT has a capability to assess those subtle changes whereas traditional diagnosis requires longer observation time [37]. In addition to the mucosa thickness, OCT has been used to quantify airway volume [33]. The degree of airway obstruction and edema can be correlated with the airway volume. Furthermore, temporal changes in the airway volume can be utilized to estimate airway compliance which relates to the mechanical properties [40-42]. In this section, we will summarize the previously conducted OCT studies related to inhalation injury.

### Sulfured mustard

**Figure 2:**
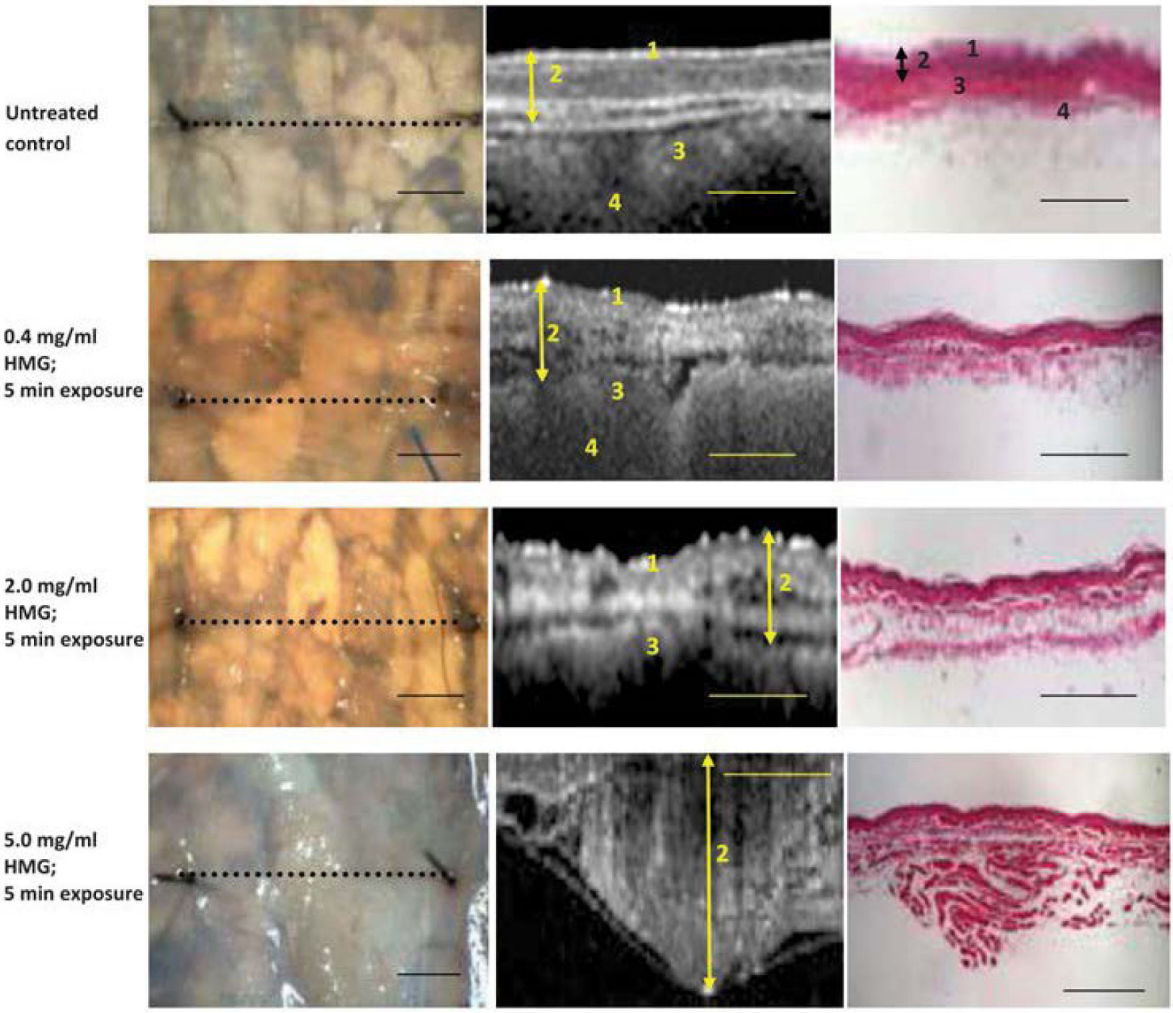
Changes in the tissue structure after exposure to different concentrations of Half mustard gas (HMG). Extensive tissue response, such as blistering, membrane opacity, and broken vessels, were observed in the HGM model (1: keratinized surface layer; 2: flat stratified squamous layer; 3: submucosa; 4: smooth muscle). Scale bar: 1mm. Reprinted with permission [43].

Sulfured mustard is a vesicant (blistering) agent and can cause significant tissue damage, leading to obstruction and edema. Hammer-Wilson et al. first demonstrated the potential use of OCT in detecting tissue damage due to exposure to 2-chloro-ethyl-ethyl-sulfide, known as half mustard, using a hamster cheek model [43]. OCT was able to detect morphological changes in the mucosa and muscular layer that were not visible on gross visual examination such as bronchoscopy (Figure 2). Kreuter et al. then reported the changes in the airway epithelium and mucosa *in vivo* in a ventilated rabbit exposed to half mustard [44]. The endoscopic OCT probe was placed at the distal portion of the trachea. The changes in the epithelium were apparent within a few minutes after exposure in OCT images, and signs of epithelium detachment and haemorrhage continued to develop over the next few hours.

### Methyl isocyanate (MIC)

**Figure 3:**
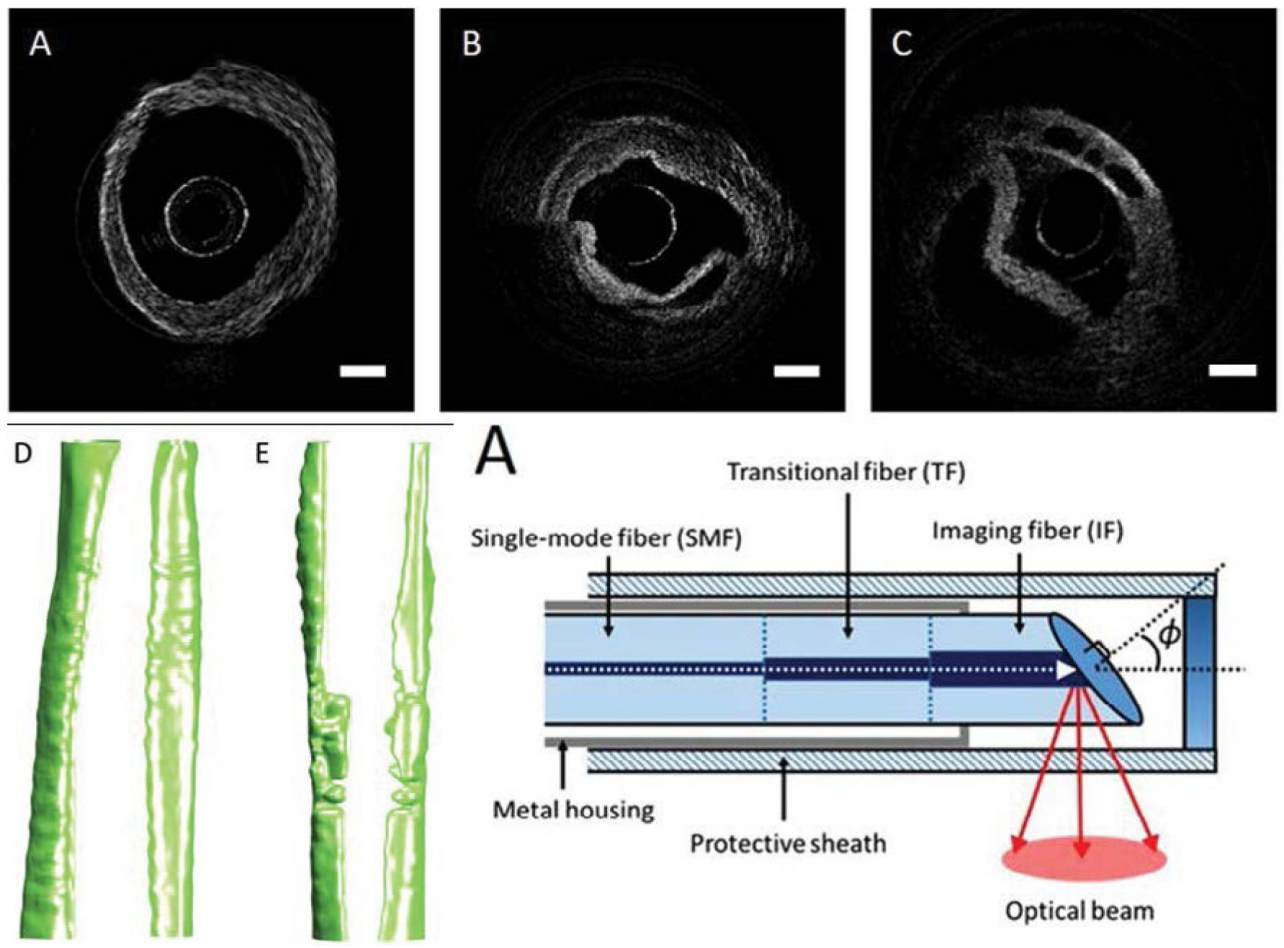
OCT images and 3D reconstruction of rat trachea exposed to MIC. OCT endoscopic images of (A) healthy rat airway and (B-C) MIC exposed airway. (D) Airway reconstruction of healthy rat and (E) MIC exposed rat. (F) A miniature fiber endoscopic probe with 0.4 mm diameter was designed for rat airway imaging. Scale bar: 0.5 mm. Reprinted with permission from ref. [33].

MIC is an industrial by-product. Accidental release of MIC in Bhopal, India killed thousands of civilians, which is considered one of the worst industrial disasters in history [45]. Inhalation of the gas causes airway edema and epithelium delamination, leading to substantial airway obstruction. The mechanism of MIC has been studied in mice [46] and rats [47], but no effective rescue agents have been developed. Miao et al., first demonstrated that the degree of airway obstruction can be quantified in a rat exposed to MIC using a miniature OCT endoscope [33]. Combined with automated segmentation technique based on graph theory, OCT provides capabilities for rapid assessment of airway structure, volume, site of maximum airway constriction, and aerodynamic characteristics (Figure 3).

### Smoke inhalation injury

Smoke inhalation injury is the leading cause of death in fire victims. In modern burn care, diagnosis of inhalation injury has been a particularly challenging yet an important problem. Inhalation injury complicates the burn and increase mortality and morbidity for up to 20% of burn patients [7]. Numerous studies have used endoscopic OCT in combination with a flexible bronchoscope to assess early changes in the tissue histopathology and predict the outcome [36-39,48]. Most of those studies focus on the quantification of mucosa and airway epithelial thickness after exposure to cold smoke. Brenner et al. reported that early tissue changes, such as hyperemia and edema, observed during *in vivo* OCT imaging were lost during the histological preparation, indicating the importance of a non-invasive, intra-operative imaging technique [38].

## Characterization Techniques for Airway Injury and Recovery

Typical OCT imaging provides structural information of airway tissue with 3-10 um spatial resolution. To automate the analysis, post-processing techniques have been developed for OCT airway images to identify and characterize different layers of tissue. Additionally, several techniques have been used to obtain functional information from OCT, such as airway compliance [41,42], birefringence property [49], and cilia beating frequency [50,51].

### Automatic tissue identification and characterization

**Figure 4:**
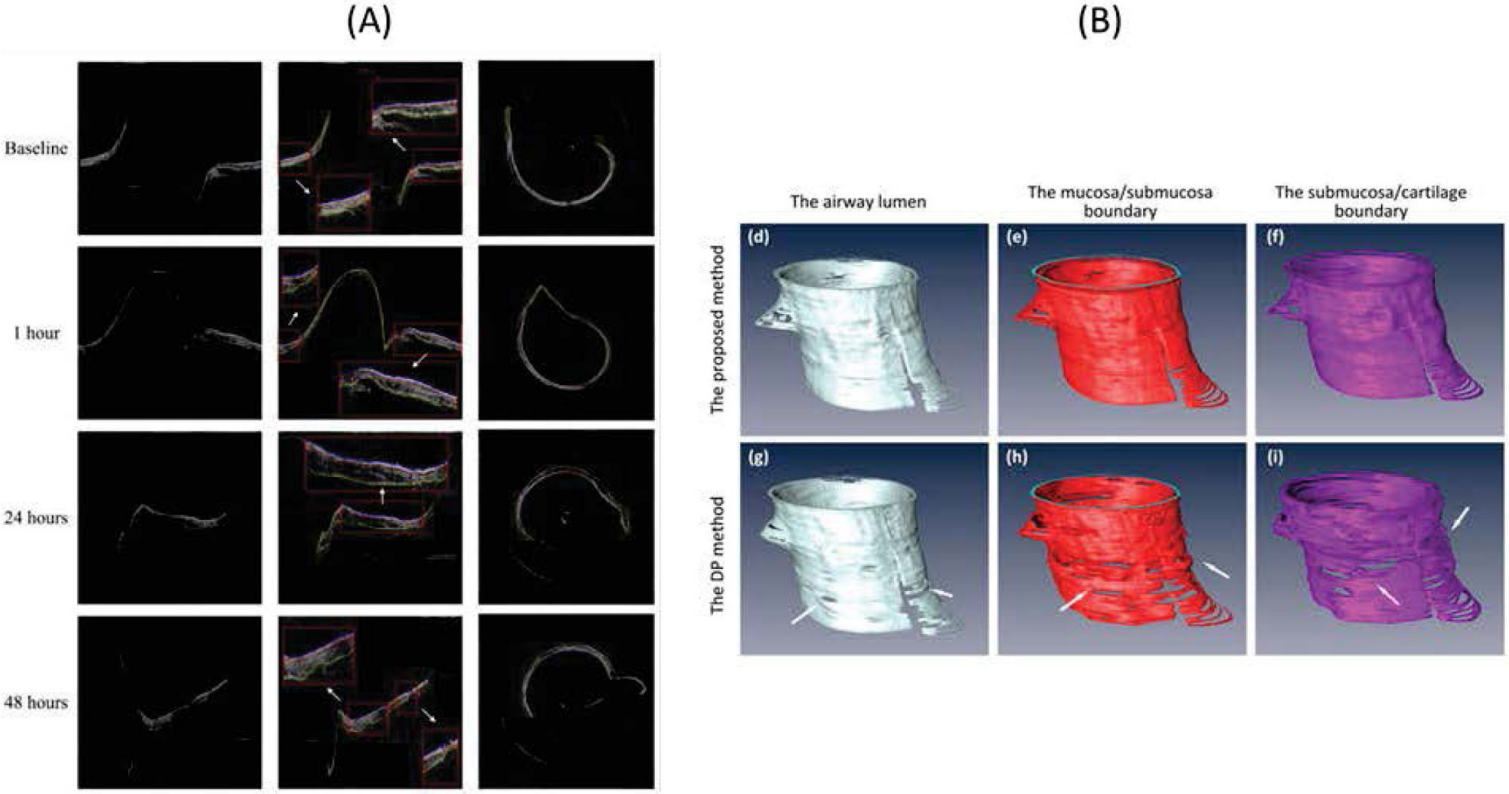
Automated tissue segmentation of sheep airway. (A) Mucosa and submucosa layer in airway can be delineated after smoke inhalation [52]. (B) 3D reconstruction of airway lumen structure and tissue layer thickness. Reprinted with permission from ref. [55].

Automated segmentation and analysis algorithms have been developed by several groups to assist data interpretation for airway OCT. From airway OCT images, we can automatically obtain information of tissue layer thickness [52,53], mucus secretion [54], and airway dimension [33,55]. Li et al., proposed a robust segmentation algorithm based on graph theory to delineate boundaries between mucosa, submucosa, and cartilage and quantified airway thickness changes during smoke inhalation using a sheep model [52]. Increase in both mucosa and submucosa layer thicknesses were observed using automated analysis which matches well with manually segmented results (Figure 4). The algorithm can be extended to reconstruct 3D airway structure and create a virtual bronchoscopic view to visualize airway narrowing [55]. Using a similar graph theory-based algorithm, Miao et al., performed a 3D reconstruction of a rat airway exposed to MIC gas [33].

### Compliance and birefringence measurement

There is a currently limited understanding of the kinetics of airway repair process after inhalation injury. The airway may not be restored to the original structure and function after recovering from inhalation injury [56]. Studies show that patients exposed to chlorine gas continued to experience symptoms, such as fibrosis, hyperplasia, airway hyper responsiveness, after recovery [57,58]. Therefore, a non-invasive visualization tool is needed to provide information on tissue components, such as elastin, collagen content, and smooth muscle thickness during the repair process. A couple of studies monitored mechanical properties and smooth muscle thickness in the airway thorough endoscopic OCT. Robertson et al., first reported compliance measurement of in vivo rabbit trachea using the airway deformation induced by the tidal breathing [40]. Oldenburg reported in vivo compliance measurement of porcine airway using a long-range OCT imaging system [41,42]. Since compliance is directly linked to elastin and collagen contents in the airway, the changes in the compliance can indicate abnormalities such as fibrosis and airway remodelling. Adams et al., developed an approach to visualize endoscopic smooth muscle in the airway using polarization sensitive OCT [49]. Polarization-sensitive OCT can detect birefringence signals coming from tissue with an ordered structure, such as smooth muscle fiber bundles and collagen. They observed an increase in smooth muscle thickness in an asthma patient which can be assessed during bronchial thermoplasty procedures.

### Ciliary function

**Figure 5:**
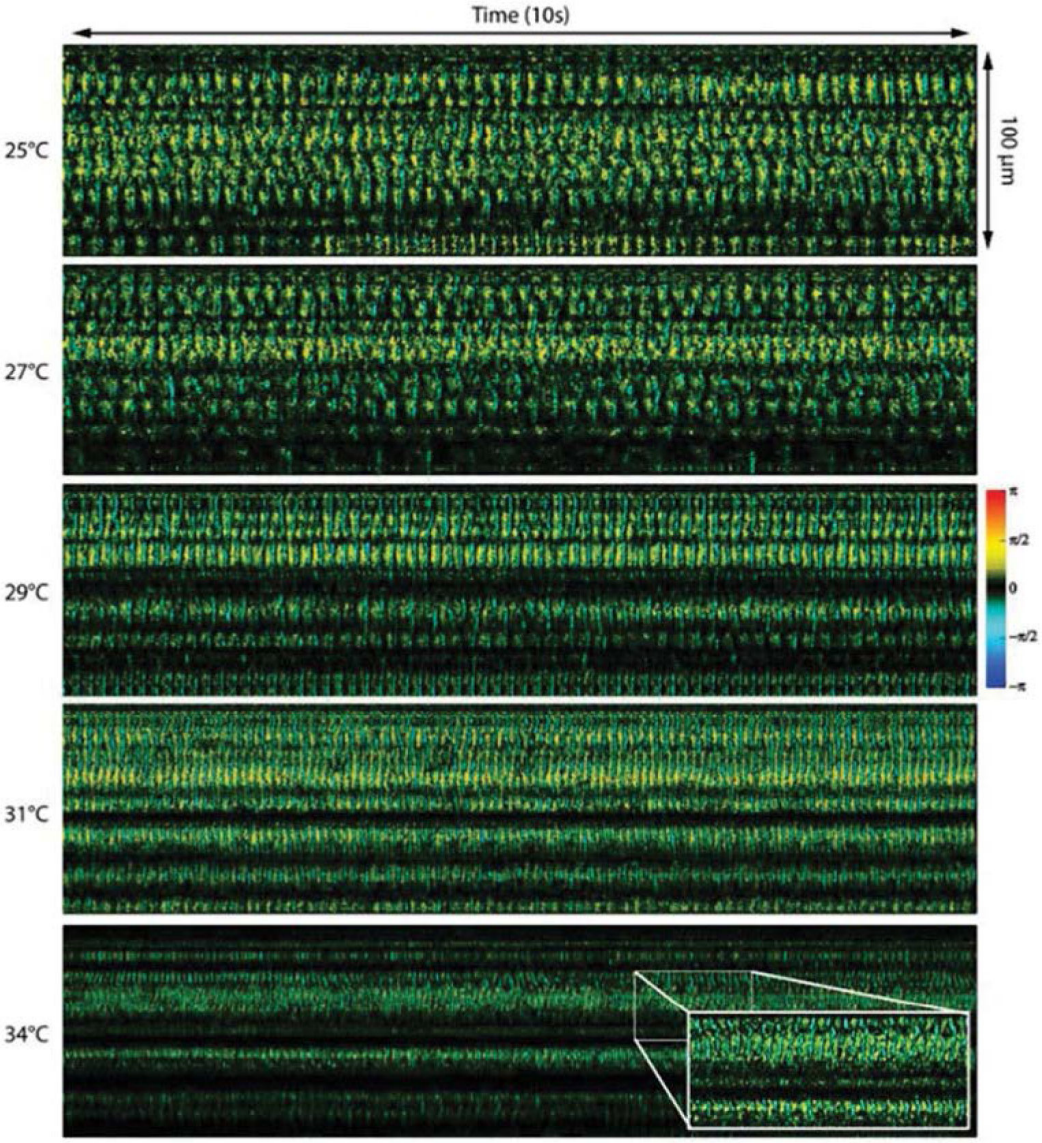
Visualization of cilia beating frequency at different temperatures using Doppler OCT. Cilia beats faster as the temperature increases. Reprinted with permission from ref. [51].

In inhalation injury, epithelium and mucociliary cells are damaged by toxins. This leads to disturbed mucociliary transportation which prevents the clearance of mucus and bacteria. Currently, real-time visualization of cilia motion cannot be achieved due to the sensitivity and spatial resolution required to visualize the cilia; the size of the bronchotracheal cilia layer is 6-7 um [59]. Liu et al., developed an ultra-high-resolution OCT system (“micro-OCT”) with 1 um axial resolution using a supercontinuum light source to visualize airway epithelial cell cilia [50]. Using a “micro-OCT system”, they were able to capture the ciliary stroke pattern for the first time. However, the supercontinuum light source is bulky and expensive compared to the light source used in standard swept-source OCT and may not be suitable for clinical settings at this time. Taking advantage of the nanometer sensitivity of Doppler OCT, Jing et al., has quantified the cilia beating frequency and visualize the temporal beating pattern of ciliary motion in a rabbit airway using a swept source laser [51]. Doppler OCT is a functional extension of OCT imaging that is capable of measuring displacements or movements in the sample with nanometer sensitivity. An increase in the beating frequency was observed as ambient temperature rises (Figure 5).

## Conclusion

Accurate assessment of the airway tissue state is important for improving the survival rate from inhalation injury and ensuring proper tissue repair. Benefiting from non-invasive, high-resolution, and high-speed imaging capability, endoscopic OCT can provide a rapid and quantitative assessment of tissue inflammation, hyperemia, and sloughing, and can be used to reconstruct a three-dimensional virtual bronchoscopy image to identify airway narrowing. In addition to structural information, compliance and birefringence signals from smooth muscle can provide important information during the healing process. Ciliary transportation is an active area of research, and endoscopic OCT has spatial resolution and sensitivity to visualize ciliary motion in vivo. In addition, endoscopic OCT is well suited for multimodal imaging since it can be easily incorporated into a working channel of a clinical flexible bronchoscope. In future studies, OCT combined with fluorescence imaging, in an endoscopic probe will be capable of providing not only structural information but also molecular information. Endoscopic OCT has great potential for diagnosis of inhalation injury and monitoring the recovery process.
